# Galectin-3 does not interact with RNA directly

**DOI:** 10.1093/glycob/cwad076

**Published:** 2023-10-10

**Authors:** Egan L Peltan, Nicholas M Riley, Ryan A Flynn, David S Roberts, Carolyn R Bertozzi

**Affiliations:** Department of Chemical and Systems Biology, Stanford University School of Medicine, 269 Campus Drive CCSR 4145 Stanford, CA 94305, United States; Sarafan ChEM-H, Stanford University, Stanford ChEM-H Building 290 Jane Stanford Way Stanford, CA 94305, United States; Sarafan ChEM-H, Stanford University, Stanford ChEM-H Building 290 Jane Stanford Way Stanford, CA 94305, United States; Department of Chemistry, Stanford University, 333 Campus Drive Stanford, CA 94305, United States; Stem Cell Program and Division of Hematology/Oncology, Boston Children’s Hospital, 1 Blackfan Circle, Boston, MA 02445, United States; Department of Stem Cell and Regenerative Biology, Harvard University, 7 Divinity Ave, Cambridge, MA 02138, United States; Sarafan ChEM-H, Stanford University, Stanford ChEM-H Building 290 Jane Stanford Way Stanford, CA 94305, United States; Department of Chemistry, Stanford University, 333 Campus Drive Stanford, CA 94305, United States; Sarafan ChEM-H, Stanford University, Stanford ChEM-H Building 290 Jane Stanford Way Stanford, CA 94305, United States; Department of Chemistry, Stanford University, 333 Campus Drive Stanford, CA 94305, United States; Howard Hughes Medical Institute, Stanford University, 279 Campus Drive Room B202 Stanford, CA 94305-5323, United States

**Keywords:** GALECTINS, Galectin-3, hnRNPA2B1, RNA-binding proteins

## Abstract

Galectin-3, well characterized as a glycan binding protein, has been identified as a putative RNA binding protein, possibly through participation in pre-mRNA maturation through interactions with splicosomes. Given recent developments with cell surface RNA biology, the putative dual-function nature of galectin-3 evokes a possible non-classical connection between glycobiology and RNA biology. However, with limited functional evidence of a direct RNA interaction, many molecular-level observations rely on affinity reagents and lack appropriate genetic controls. Thus, evidence of a direct interaction remains elusive. We demonstrate that antibodies raised to endogenous human galectin-3 can isolate RNA-protein crosslinks, but this activity remains insensitive to *LGALS3* knock-out. Proteomic characterization of anti-galectin-3 IPs revealed enrichment of galectin-3, but high abundance of hnRNPA2B1, an abundant, well-characterized RNA-binding protein with weak homology to the N-terminal domain of galectin-3, in the isolate. Genetic ablation of *HNRNPA2B1*, but not *LGALS3*, eliminates the ability of the anti-galectin-3 antibodies to isolate RNA-protein crosslinks, implying either an indirect interaction or cross-reactivity. To address this, we introduced an epitope tag to the endogenous C-terminal locus of *LGALS3*. Isolation of the tagged galectin-3 failed to reveal any RNA-protein crosslinks. This result suggests that the galectin-3 does not directly interact with RNA and may be misidentified as an RNA-binding protein, at least in HeLa where the putative RNA associations were first identified. We encourage further investigation of this phenomenon employ gene deletions and, when possible, endogenous epitope tags to achieve the specificity required to evaluate potential interactions.

## Introduction

Prior targeted and untargeted reports have identified members of the Galectin family as possible RNA binding proteins (Wang et al. 1995; Castello et al. 2012; Huang et al. 2018; Queiroz et al. 2019). Commonly understood to function on the cellular surface and in the extracellular matrix (Kuwabara et al. 2003; Vasta 2009; Ruvolo 2016; Thiemann and Baum 2016), galectins lack signal sequences, are synthesized on cytosolic ribosomes (Cho and Cummings 1995), and secreted through unconventional secretion mechanisms (Roff and Wang 1983; Cooper and Barondes 1990; Lindstedt et al. 1993; Sato et al. 1993; Mehul and Hughes 1997; Bänfer et al. 2018; Popa et al. 2018; Zhang et al. 2020; Davuluri et al. 2021). Cytosolic galectins are understood to sense and respond to endolysosomal membrane damage (Aits et al. 2015; Jia et al. 2018; Jia, Bissa et al. 2020a; Jia, Claude-Taupin et al. 2020b). The association between galectins and RNA was postulated in the early 1990s after the discovery of weak homology between the intrinsically disordered N-terminal domain of galectin-3 and members of the hnRNP series of RNA binding proteins (RBPs) (Jia and Wang 1988). This report was followed by a series of studies identifying galectins in the nucleus (Seve et al. 1985; Moutsatsos et al. 1986; Moutsatsos et al. 1987; Wang et al. 1991: 198; Sève et al. 1993; Hubert et al. 1995; Vyakarnam et al. 1998) and suggesting a role for galectin-3 in pre-mRNA splicing (Dagher et al. 1995; Wang et al. 1995; Vyakarnam et al. 1998; Park et al. 2001; Wang et al. 2006; Gray et al. 2008; Haudek et al. 2009; Voss et al. 2012; Patterson et al. 2015; Haudek et al. 2016). Further, recent RBP screens have identified galectin-3 as a candidate RNA binding protein (Castello et al. 2012; Huang et al. 2018; Queiroz et al. 2019).

However, unlike most RNA-binding proteins, galectin-3 lacks a canonical RNA-recognition motif suggesting a non-canonical mode of interaction (LGALS3 - *Homo sapiens* (Human) | UniProt). The potential RNA-binding function of galectins could represent a tantalizing link between cell-surface glycobiology and nuclear RNA biology, especially in light of recently identified cell-surface glycoRNAs (Flynn et al. 2021).

To investigate the possibility of a direct interaction between galectin-3 and RNA, we pursued an UV-Crosslinking and Immunoprecipitation (irCLIP) approach leveraging zero-distance UV-crosslinking, an infrared-dye-conjugated and biotinylated ligation adaptor, and commercial anti-galectin-3 antibodies to isolate RNA-protein crosslinks (Zarnegar et al. 2016). Here, we show that while capture of endogenous galectin-3 can generate irCLIP signal, genetic ablation of *LGALS3* does not eliminate this RNAse-sensitive signal. Further, we show that some commercial anti-galectin-3 antibodies cross-react with well-characterized RNA-binding proteins and deletion of one of these RNA-binding proteins, *HNRNPA2B1*, eliminates the observed galectin-3 RNA-association. Finally, we use CRISPR-Cas9 gene editing (Jinek et al. 2012; Lin et al. 2014; Cho et al. 2022) to insert an epitope tag, demonstrating that galectin-3 does not associate with RNA directly.

## Materials and methods

### Cell culture

ATCC reference HeLa (ATCC CCL-2) and HEK-293 T (ATCC CRL-1573) cells were passaged in DMEM (Gibco 11965092) with 10% FBS in the absence of antibiotics and frozen in complete growth medium +10% DMSO (Sigma). All cell lines were maintained at 37 °C and 5% CO2. Cells were routinely tested for mycoplasma using Lonza MycoAlert Plus (Lonza) and a PCR-based test (Uphoff and Drexler 2005).

### CRISPR-Cas9 editing

Three non-overlapping gRNAs targeting a conserved early exon were selected using Synthego’s CRISPR Design Tool and synthesized as modified sgRNAs (Synthego). For each clone, gene knock-out was verified at the DNA level with sanger sequencing (Elim Bio, Hayward, CA) and at the protein level by immunoblot. See Supplementary Methods for additional detail.

### Endogenous tag

Based on the cell engineering pipeline from OpenCell (Cho et al. 2022). See Supplementary Methods for additional detail.

### irCLIP

See Supplementary Methods for details of *Adaptor synthesis*, *UV Crosslinking, Sub-cellular fractionation*., *Immunoprecipitation, and Adaptor Ligation.* Following ligation elution, the total volume of the sample was analyzed SDS-PAGE with an Odyssey CLx Imager (LI-COR), visualizing ligated RNA in the 800 channel. Target proteins run ~15 kDa above expected MW (RNA fragment+preA-L3-800 adaptor). See Supplementary Methods for additional details.

### Mass-spectrometry

*MS Data Acquisition.* See Supplementary Methods. The same MS method was used for CLIP-MS, GeLC-MS/MS, and IP-MS experiments. *MS Data Analysis.* Peptide spectral matches were made against a target-decoy human reference proteome database downloaded from Uniprot (Elias and Gygi 2007). For quantitative comparisons, Peptides were identified with MaxQuant label-free quantitation. Relative enrichment of log2-transformed intensities was assessed on a per-protein basis with an FDR computed by a Benjamini-Hochberg adjusted t-test. Peptides were analyzed on a per-protein basis and plotted and annotated using the ggplot2 package in R (Wickham 2009).

## Results

### Anti-galectin-3 antibodies recognize RNA-crosslinked proteins in irCLIP

**Fig. 1:**
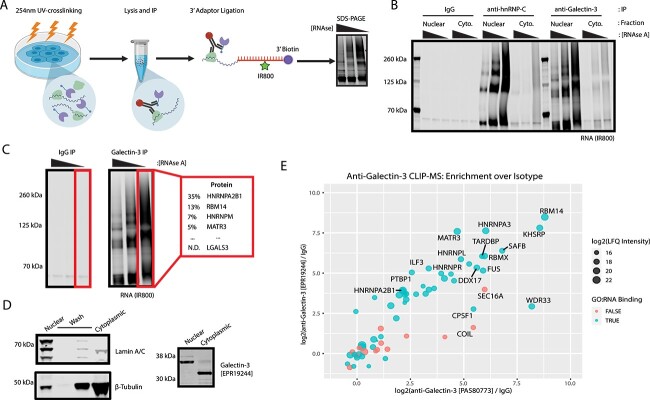
A) irCLIP workflow adapted from Zarnegar et al. 2016 (Zarnegar et al. 2016). B) irCLIP in nuclear and cytosolic fraction of HeLa. Anti-hnRNPA2B1 and anti-Galectin-3 identify RNA primarily in the nuclear fraction. C) irCLIP-MS in-gel digest and proteomic identification of proteins associated with irCLIP signal. Galectin-3 is not detected in the region of the gel containing RNA signal. D) Fractionation of HeLa cells. Demonstration of separation of nuclei (Lamin A/C) from cytosol (Tubulin). Anti-Galectin-3 mAb [EPR19244] detects bands in both the cytoplasmic and nuclear fractions. E) Enrichment profiles of two anti-galectin-3 antibodies by IP-MS. Both antibodies enrich well characterized RNA-binding proteins.

To probe for an *in cellulo* interaction between galectin-3 and RNA, we pursued an irCLIP approach (Fig. 1A). In this approach, the target galectin protein is isolated from UV-crosslinked cell lysate and partially digested with RNAse to enable ligation of a pre-adenylated DNA adaptor with T4 RNA ligase (Zarnegar et al. 2016). We assessed a series of anti-galectin-3 antibodies for their ability to isolate an RNA-protein crosslinks. Anti-galectin-3 antibodies isolated protein crosslinks from a variety of oncogenic cell lines (Fig. S1, Fig. S2). Intriguingly, multiple commercial anti-galectin-3 antibodies demonstrated RNA signal in irCLIP SDS-PAGE gel assays (Fig. 1B). Furthermore, sub-cellular fractionation of UV-crosslinked cells revealed that the anti-galectin-3 irCLIP signal localized to the nuclear fraction, consistent with prior reports of nuclear localization of galectin-3 (Fig. 1C) (Seve et al. 1985; Moutsatsos et al. 1986; Moutsatsos et al. 1987; Wang et al. 1991: 198; Sève et al. 1993; Hubert et al. 1995; Vyakarnam et al. 1998). However, the nuclear band recognized by anti-galectin-3 antibodies appeared ~8 kDa above the cytosolic band recognized by the same antibody, suggesting a possible post-translational modification or off-target reactivity (Fig. 1D).

### Anti-galectin-3 antibodies isolate RNA-binding proteins

To evaluate the specificity of the anti-galectin-3 irCLIP, which is performed under high salt washes usually enabling stringent isolation of target proteins, we performed in-gel digest of proteins associated with RNA signal. Surprisingly, using mass spectrometry, we did not detect any galectin-3 peptides overlapping with the RNA-associated signal (Fig. 1C). Rather, the most abundant proteins associated with the RNA signal were nuclear splicing factors, including the highly abundant hnRNPA2B1 (Fig. 1E, Fig. S3). hnRNPA2B1 has been observed as a putative interactor of galectin-3, in another anti-galectin-3 co-IP experiment (Fritsch et al. 2016). IP-MS with anti-galectin-3 in cell fractions confirms enrichment of galectin-3 in addition to splicing factors, including hnRNPA2B1 (Fig. S4). While there is little structural homology between hnRNPA2B1 and galectin-3, both proteins possess an intrinsically disordered domain rich in prolines and tyrosines, with semi-regular spacing (Fig. S5) (Lin et al. 2017; Martin et al. 2020). Additionally, as tyrosine commonly forms crosslinks with nucleobases following UV-irradiation, their abundance should improve crosslinking efficiency if galectin-3 binds RNA directly (Kunkel et al. 1981; Stützer et al. 2020). Therefore, the proposed interaction is either indirect in nature or a result of off-target binding of classical RNA-binding proteins hnRNPA2B1 by the anti-galectin-3 antibodies. Specificity issues of commercial affinity reagents have been well documented with estimates suggesting ~50% of commercial antibodies recognize the wrong target (Berglund et al. 2008; Bradbury and Plückthun 2015).

### Loss of hnRNPA2B1, but not galectin-3, depletes irCLIP signal

**Fig. 2:**
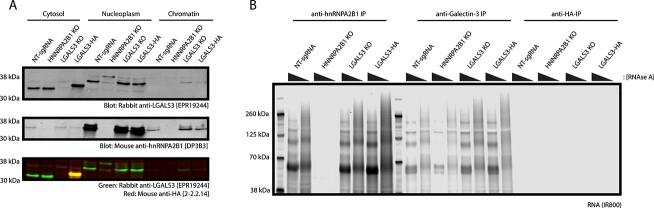
A) Sub-cellular fractionation of HeLa following CRISPR KO or endogenous tagging of *LGALS3.* Loss of HNRNPA2B1 eliminates the nuclear band detected by the anti-galectin-3 mAb [EPR19244]. Loss of LGALS3 does not impact the nuclear band detected by anti-galectin-3 mAb [EPR19244]. B) irCLIP of HeLa following CRISPR KO and endogenous tag. RNA signal is preserved in the anti-galectin-3 irCLIP in the *LGALS3* KO background, but lost in the *HNRNPA2B1 KO* background. Anti-HA irCLIP fails to produce any RNA signal in the endogenously tagged LGALS3-HA background.

To control for off-target binding of the commercial affinity reagents, we used a multi-guide RNP-based CRISPR-Cas9 editing approach to knock-out expression of galectin-3 and hnRNPA2B1 from HeLa cells. Following CRISPR-KO and clonal selection by limiting dilution, we verified the loss of galectin-3 and hnRNPA2B1 expression by immunoblot (Fig. S6). In the *LGALS3* KO background, anti-galectin-3 irCLIP retains the associated RNA signal. However, anti-galectin-3 irCLIP in the *HNRNPA2B1* KO background does not identify the associated RNA signal, suggesting that galectin-3 does not bind RNA directly and may not associate with RNA at all (Fig. 2B).

### irCLIP of endogenously tagged galectin-3 does not isolate RNA-protein crosslinks

To test this directly, we introduced an HA-tag to the endogenous LGALS3 locus at its C-terminal end. In characterizing RNA-binding proteins, endogenous tags are preferred to maintain the endogenous expression levels and preserve native RNA-binding patterns as many of these low-affinity high-valency RNA-protein interactions are highly sensitive to context and concentration (Ule et al. 2018; Alberti et al. 2019; Hafner et al. 2021). Further, this tag lets us test if the postulated galectin-3-RNA interaction is direct or an artifact of antibody cross-reactivity. Using CRISPR-Cas9 mediated HDR-directed editing, we inserted an HA epitope tag to the C-terminal region of *LGALS3,* without disrupting the 3’UTR (Lin et al. 2014; Feng et al. 2017; Cho et al. 2022). HA-tagged galectin-3 retains glycan binding activity (Fig. S10) and nucleocytosolic localization via IF (Fig. S11). Rather conclusively, the anti-HA IP isolates HA-tagged galectin-3, but an anti-HA irCLIP in the HA-tagged galectin-3 background identified no irCLIP signal above the non-tagged control (Fig. 2B). In addition, an orthogonal physical-chemical method for isolating RNA-protein crosslinks (Fig. S7) also failed to identify a direct interaction between galectin-3 and RNA (Figs S8, S9) (Queiroz et al. 2019; Villanueva et al. 2020). Therefore, the association of galectin-3 with RNA is likely not direct and should be reevaluated in light of observed mAb reactivity.

### Some anti-galecitn-3 mAbs enrich known RPBs via IP-MS in *LGALS3* KO HeLa

To assess the specificity of selected galectin-3 mAbs, rat anti-LGALS3 [Mac2] and mouse anti-LGALS3 [A3A12], previously used to study galectin-3 (Liu et al. 1996; Vyakarnam et al. 1998; Gray et al. 2008)**.** We assayed the specificity of these mAbs, and the rabbit anti-LGALS3 [EPR19422], by IP-MS to characterize their enrichment in the presence and absence of galectin-3. IP-MS of the selected mAbs revealed enrichment of known, well-characterized RBPs in both the NT-sgRNA and *LGALS3* KO backgrounds (Figs S12–S15).

## Discussion

The context-dependent nature of many RNA-protein interactions makes proving non-interaction a Sisyphean task (Castello et al. 2012; Kramer et al. 2014; Hentze et al. 2018; Trendel et al. 2019; Backlund et al. 2020; Huppertz et al. 2022; Perez-Perri et al. 2023). Validation of candidate RBPs is essential. Galectin-3 in appeared in multiple RBP screens, co-isolates with RNA-protein crosslinks, may participate pre-mRNA maturation, yet fails to bind RNA directly when assessed under stringent conditions.

This study does not, and cannot, rule out the possibility of a non-classical RNA binding function for other galectins in other contexts. Investigators should proceed with caution and include positive (canonical RBPs) and negative (genetic deletions) controls for future *in cellulo* exploration of putative galectin-RNA interactions. As many RBPs not only self-associate, but also associate with other RBPs, attribution of RNA interactions to a target RBP requires comparison to an RBP-depleted sample (e.g. genetic deletion) or demonstration of the exclusion of other RBPs (e.g. proteomics).

While this investigation used high stringency methods to probe for direct RNA-protein interactions, indirect but functional interactions may exist. Reports proposing indirect interactions use non-zero distance formaldehyde crosslinking to capture these interactions (Coppin et al. 2017). However, recent investigations of the RNA-binding properties of galectins discover phenotypic evidence suggesting RNA binding and thus infer an RNA-protein interaction, but often lack direct evidence in vitro or *in cellulo* (Coppin et al. 2017; Wei et al. 2021). At minimum, RNA interaction claims would require comparison to enrichment in a knock-out background, proteomic characterization of enrichments, or an epitope tag enabling antibody specificity would be necessary for causal interpretation of the contribution of a galectin to RNA binding. Without controlling for the specificity of the enrichment, it remains possible that the RNA fragments identified in an RNA-IP are not sensitive to genetic deletion, as was the case with anti-galectin-3 in HeLa. Further, high-sequencing depth (sensitivity) in workflows with high variability and few replicates, especially in non-blinded experiments as exploratory RNA-interaction experiments often are, can create non-meaningful yet statistically-significant differences through overpowered hypothesis testing of small variations in transcripts across samples. We recommend carefully assessing effect sizes and employing nonparametric statistical testing, such as bootstrapping, to control for stochastic and batch variability in untargeted, highly-powered RNA-IP sequencing studies (Kulkarni et al. 2022).

## Supplementary Material

Revised_SI_Galectin_3_does_not_directly_interact_with_RNA_V2_cwad076

Galectin_3_TableSI_Antibodies_Oligos_HDR_cwad076

Peltan_et_al_2023_MS_data_full_cwad076

## Data Availability

All relevant data and the raw mass spectrometry protein identifications are included in the online version of this article.

## CRediT author statement

Egan Peltan (Conceptualization [lead], Data curation-Lead, Formal analysis [lead], Funding acquisition [supporting], Investigation [lead], Methodology [equal], Project administration [equal], Supervision [equal], Validation [lead], Visualization [lead], Writing—original draft [lead], Writing—review & editing [equal]), Nicholas M. Riley (Data curation [supporting], Funding acquisition [supporting], Investigation [supporting], Methodology [supporting], Software [equal], Writing—review & editing [equal]), Ryan A. Flynn (Methodology [supporting], Resources [supporting], Supervision [supporting], Writing—review & editing [supporting]), David Roberts (Data curation [supporting], Investigation [supporting], Writing—review & editing [supporting]), Carolyn Bertozzi (Conceptualization [supporting], Funding acquisition [lead], Project administration[supporting], Writing—review & editing [supporting])
