# Correction to: Galectin-3 does not interact with RNA directly

**DOI:** 10.1093/glycob/cwae027

**Published:** 2024-03-27

This is a correction to: Egan L Peltan, Nicholas M Riley, Ryan A Flynn, David S Roberts, Carolyn R Bertozzi, Galectin-3 does not interact with RNA directly, *Glycobiology*, Volume 34, Issue 1, January 2024, cwad076, https://doi.org/10.1093/glycob/cwad076

In the originally published version of this article, there was an error in Figure 2A, whereby the labels LGALS3 KO and HNNRPA2B1 KO were transposed.

The Publisher apologizes for not correcting this at an earlier production stage.

The article has now been updated with the correct version of the Figure.

