# Supplementary material for: Galectin-3 does not interact with RNA directly
Source: Glycobiology. 2023 Oct 10;34(1):cwad076. doi: 10.1093/glycob/cwad076 (PMC11648975; doi:10.1093/glycob/cwad076)
Supplement: Revised_SI_Galectin_3_does_not_directly_interact_with_RNA_V2_cwad076 [file revised_si_galectin_3_does_not_directly_interact_with_rna_v2_cwad076.pdf]

Supplementary Information For

Galectin-3 does not directly interact with RNA directly

Egan L. Peltan[1,5], Nicholas M. Riley[2,5], Ryan A. Flynn[3,4], David S. Roberts[2,5], Carolyn R. Bertozzi[2,5,6]

1. Department of Chemical and Systems Biology, Stanford University School of Medicine, Stanford, CA, USA 94305
2. Department of Chemistry, Stanford University, Stanford, CA, USA 94305
3. Stem Cell Program, Boston Children's Hospital, Boston, MA, USA 02115
4. Department of Stem Cell and Regenerative Biology, Harvard University, Cambridge, MA, USA 02138
5. Sarafan ChEM-H, Stanford University, Stanford, CA, USA 94305
6. Howard Hughes Medical Institute, Stanford University, Stanford, CA 94305, USA
7. Corresponding Author

MATERIALS AND METHODS:

Cell Culture

ATCC reference HeLa (ATCC CCL-2), HEK-293T (ATCC CRL-1573), MDA-MB-231 (ATCC HTB-26), and MRC-5 (ATCC CCL-171) cells were passaged in DMEM (Gibco 11965092) with 10% FBS in the absence of antibiotics and frozen in complete growth medium + 10% DMSO (Sigma). 4T1 (ATCC CRL-2539) were passaged in RPMI-1640 (Gibco 11875093) with 10% FBS in the absence of antibiotics and frozen in complete growth medium + 10% DMSO. All cell lines were maintained at 37°C and 5% CO2. Cells were routinely tested for mycoplasma using Lonza MycoAlert Plus (Lonza) and a PCR-based test.(Uphoff and Drexler 2005)

CRISPR-Cas9 Editing

Three non-overlapping gRNAs targeting a conserved early exon were selected using Synthego's CRISPR Design Tool and synthesized as modified sgRNAs (Synthego). The sgRNAs were resuspended to 100 µM in nuclease-free 10 mM Tris-EDTA pH 8.0 and complexed with 20 µM *S. py* Cas9 (Synthego) *in vitro* with a 9:1 molar ratio of sgRNA:Cas9 for 30 min prior to nucleofection. A suspension of HeLa cells was nucleofected with program CN-114 using an Amaxa 4D-Nucleofector X with SE Cell Line 4D X Kit S 32 RCT (Lonza V4XC-1032). Reactions were split between two 24-well plates and grown in complete media three days. DNA was extracted and amplified for analysis of CRISPR editing using the Synthego ICE tool. Then, from populations with >70% gene knock-out, clones were selected by limiting dilution in 96 well format. For each clone, gene knock-out was verified at the DNA level with sanger sequencing (Elim Bio, Hayward, CA) and at the protein level by immunoblot.

Endogenous Tag

Based on the cell engineering pipeline from OpenCell (Cho et al. 2022), we used HDR-based strategy to incorporate the tag at the endogenous C-terminal locus of *LGALS3*. We designed a synthetic HDR template (IDT) with 40bp of flanking homology to introduce the tag. To induce a dsDNA break, we selected a high-efficiency CRISPR guide adjacent to the stop codon. To promote HDR, we induced a G2/M arrest with 200 ng/mL Nocodazole (ApexBio, A8487) 16h prior to nucleofection.(Lin et al. 2014) Finally, *S. pyogenes* Cas9 RNPs were pre-assembled *in vitro*, mixed with ssDNA HDR donor

template, and delivered into HeLa cells using an Amaxa 4D-Nucleofector X with SE Cell Line 4D X Kit S 32 RCT (Lonza V4XC-1032) with program CN-114. Reactions were split between two 24-well plates and grown in complete media for three days. DNA was extracted and amplified for analysis of CRISPR editing using the Synthego ICE tool.

irCLIP

Adaptor synthesis. The preA-L3-irCLIP adaptor was constructed as in Zarnegar 2016 (Zarnegar et al. 2016). The L3-Azide-Biotin oligonucleotide (5′-OH-AGATCGGAAGAGCGGTTCAGAAAAAAAAAAAA/iAzideN/AAAAAAAAAAAA/3Bio/-3′) was synthesized at the 1 µmol synthesis scale (approximate yield of 25 nmol) by Integrated DNA Technologies (IDT, Coralville, IA, U.S.). The non-standard modifications are an internal Azide at position 32 (/iAzide/) and the 3' biotin modification (/3bio/) blocking the 3' end. In a 1.5 mL microcentrifuge tube, 10 nmol of oligonuclotide was phosphorylated with 250 units of T4 PNK (NEB, M0201S) at 1 mM ATP in a reaction volume of 250 µL for 1 h at 37°C. The total reaction volume was precipitated by adding 20 µL of 3 M NaOAc pH 5.5 to the reaction, mixing well with a pipette, and addition of 0.75 mL 100% ethanol at -20°C. The reaction was incubated at -20°C for 30 min and then the phospho-oligonucleotide was pelleted for 30 min at 18,000 x g in a 4°C table-top microcentrifuge. The supernatant was removed from the phospho-oligonucleotide pellet. Then, the pellet was washed one time with -20°C 80% ethanol, air dried at ambient temperature for 10 min, and then resuspended in 250 µL of nuclease-free water. The entire volume of the phospho-oligonuclotide was pre-adenylated using the 5′ DNA Adenylation Kit (NEB E2610L) by addition of 37.5 µL each of 1 mM ATP and 10x buffer with 50 µL of Mth RNA ligase. The reaction was incubated for 2 h at 65°C before inactivation at 85°C for 10 min. 40 µL of 3 M NaOAc pH 5.5 was added to the total reaction volume (375 µL), mixed well with a pipette, and precipitated overnight at -20°C following the addition of 1 mL of 100% ethanol. The precipitated preA-oligonucleotide was pelleted for 30 min at 18,000 x g for 30 min at 4°C. The supernatant was removed, the pellet was washed one time with -20°C 80% ethanol, air dried at ambient temperature for 10 min, and then resuspended in 180 µL of 1x PBS pH 7.4. For the click-chemistry conjugation, 20 µL of 10 mM AZDye-800-DBCO (Click Chemistry Tools, #1564-5) in DMSO was added to the preA-oligonucleotide solution and incubated for 2

h at 37°C. The 200 µL click reaction was purified by addition of 19x volumes of Zymo DNA Binding Buffer (Zymo Research, D4004-1-L), aliquoted evenly across 20 Zymo-Spin IIN Columns (Zymo Research, C1019-250), and spun at 6,000 x g for 30s. The columns were washed twice with 0.3 mL of fresh 80% ethanol followed by a dry spin at 6,000 x g for 2 min. Each column was transferred to a new 1.5 mL microcentrifuge tube and the preA-oligonucleotide-800 was eluted by the addition of 50 µL of nuclease-free water. Columns were incubated at ambient temperature for 2 minutes before elution by spinning for 2 min at 18,000 x g. The pale-blue eluates were pooled and quantiated by nanodrop. The adaptor was then diluted in nuclease-free water to a working stock of 1 µM and stored at -20°C indefinitely. Working stocks were freeze-thawed more than 30 times without noticeable loss of activity.

UV Crosslinking. As in Zarnegar and Flynn et al., adherent HeLa cells were grown in 10 cm plastic dishes to ~80% confluence, rinsed once with ice-cold 1x PBS pH 7.4, and crosslinked with 254 nm UV-C light at 0.3 J/cm^2 (Zarnegar et al. 2016). Immediately following UV-C crosslinking cells were either immediately lysed in ice-cold CLIP buffer (50 mM HEPES pH 7.5, 200 mM NaCl, 1 mM EDTA, 10% Glycerol, 0.1% Nonidet-P40/IGEPAL CA-630, 0.2% Triton X-100, 0.5% N-lauroylsarcosine) supplemented with 1x Mammalian ProteaseArrest™ (G Biosciences, #786-331) or lifted with 0.25% Trypsin-EDTA (Cytiva, SH3004201) for 5 min at 37°C, inactivated with 2 volumes of complete media, and pelleted for 3 min at 300g at 4°C immediately proceeding to fractionation.

Sub-cellular fractionation. Subcellular fractionation was performed as described in Gagnon et al. to isolate membrane/cytoplasm, nucleoplasm, and chromatin fractions (Gagnon et al. 2014). UV-crosslinked cells were washed once with 5 mL of ice-cold 1x PBS pH 7.4 to remove residual media and trypsin (often contaminated with RNAse activity). To isolate nuclei from the membrane/cytoplasmic fraction, a hypotonic lysis with HLB (10 mM Tris pH 7.5, 10 mM NaCl, 3 mM $MgCl_2$, 0.3% IGEPAL CA-630 (Sigma-Aldrich, #I8896-50ML), 10% glycerol, 1x Mammalian ProteaseArrest™) was performed by incubating UV-crosslinked HeLa cells in a suspension of 1.5 mL of HLB for each 10 million cells (~75 mg wet) on ice for 10 min, followed by a breif (<3 s) vortex at high speed. The nuclei were pelleted from the cell suspension for 8 min at 800g at

4°C. The cytoplasmic fraction was transferred to a 15 mL falcon tube, added 5 M NaCl to final concentration of 140 mM, and frozen on dry ice in microcentrifuge tubes. The nuclei pellet was washed 4x with equal volumes of HLB by resuspending by gentle pipetting and centrifugation at 200g for 2 min at 4°C. Nucleoplasm was isolated by resuspending the nuclei in ice-cold MWS buffer (10 mM Tris pH 7.5, 4 mM EDTA, 300 mM NaCl, 1000 mM Urea, 1% IGEPAL CA-630, 1x Mammalian ProteaseArrest™) at 0.5 mL MWS for every 75 mg or 10 million cells. Vortex at low-speed for <5 s to resuspend the nuclei. Incubate on ice for 15 min. Vortex at high-speed for >10 s to lyse the nuclei. Centrifuge at 1,000g for 5 min at 4°C to pellet the chromatin. Gently remove the supernatant (nucleoplasm) with a pipette, transfer to microcentrifuge tubes, and freeze on dry ice. Wash the chromatin pellet twice in an equal volume of MWS by vortexing, incubating on ice for 2 min, and pelleting at 500g for 3 min at 4°C. To isolate the chromatin, the pellet was resuspended in 0.5 mL of NLB (20 mM Tris pH 7.5, 150 mM KCl, 3 mM $MgCl_2$, 0.3% IGEPAL CA-630, 10% glycerol, 1x Mammalian ProteaseArrest™) for every 10 million cells, and sonicated with a Misonix S-3000 sonicator at 20% power for 45s in 15s intervals with 2 min cooling periods in-between. The sonicated suspension was then frozen on dry-ice in a microcentrifuge tube. Purity of the fractions were assessed by western blot using anti-α-Tubulin B-5-1-2 (ThermoFisher, #32-2500) and anti-Lamin-A/C (Forte/Bethyl, #A303-430A).

Immunoprecipitation. Whole-cell lysates in CLIP buffer were thawed at 4C with rotation and then briefly sonicated at 20% power for 15s in 5s intervals. Cell lysates and fractions were clarified by centrifugation at 18,000g for 15 min at 4°C. Whole cell lysates were quantitated with the Pierce BCA Protein Assay Kit in duplicate at 1:5 dilution. Whole cell lysates were normalized to 1 mg/mL in CLIP buffer for use in IP experiments. IPs comparing across fractions were diluted with 2 volumes of CLIP buffer, except cytoplasmic fractions which were diluted with 3 volumes of CLIP buffer. For adaptor ligation experiments, the lysates were pre-digested with RNAse A (ThermoFisher, EN0531) at two or three concentrations ranging from 100 ng/mL to 1 ng/mL for 10 min at 37°C, then immediately transferred to ice where 1 µL of SUPERase-In RNAse Inhibitor (ThermoFisher, AM2696) was added. Up to 5 µg of antibody was loaded onto magnetic Protein G Dynabeads (ThermoFisher, 10004D) at 30 µg Ab /100 µL Protein G

beads by incubation in 1x PBS + 0.02% Tween-20 at ambient temperature for 30 min with rotation. Mouse host primary antibodies were complexed with ½ equivalent of rabbit anti-mouse IgG Fc secondary (ThermoFisher, PI31194) whereas other host IgGs were complexed directly onto Protein G beads. An equal loading each antibody-bead complex was split evenly across lysate pre-digested with RNAse A. Proteins were immunoprecipitated overnight at 4°C with end-over-end rotation. Immunoprecipitations were centrifuged briefly at 2,000g at 4°C to collect lysate. Lysates were removed from immunoprecipitates and set aside in labeled microcentrifuge tubes. Immunoprecipitates were then washed sequentially for 10 min each at 4°C with an equal volume of High-Stringency Buffer (20 mM Tris pH 7.5, 120 mM NaCl, 5 mM EDTA, 1% Trition-X100, 1% Na-deoxycholate, 0.001% SDS), High-Salt Buffer (20 mM Tris, pH 7.5; 1 M NaCl, 5 mM EDTA, 1% Trition-X100, 1% Sodium deoxycholate, 0.001% SDS), Low-Salt Buffer (20 mM Tris pH 7.5, 5 mM EDTA) and half-volume of NT2 buffer (50 mM Tris pH 7.5, 150 mM NaCl, 1 mM $MgCl_2$, 0.0005% IGEPAL CA-630). Immunoisolates were then washed once with 0.2 mL of NT2 buffer and transferred to new microcentrifuge tubes and centrifuged at 10,000 x g for 30s to pool excess buffer for pipette aspiration. Immunoisolated RNA-protein crosslinks were then dephosphorylated with T4 PNK (NEB M0201S) for 30 min in an Eppendorf Thermomixer at 37°C, 15s 1,400 RPM, 90s rest, in a 20 µL reaction (50 mM Tris pH 7.0, 10 mM $MgCl_2$, 5 mM DTT, 20% PEG400(SigmaAldrich, 202398)) containing 7 units of T4 PNK, 0.5 units FastAP (ThermoFisher, EF065) and 0.06 µL of SUPERase-IN. Then, the T4 PNK reaction samples were cooled on ice and 3'-ligated by adding a 1/3rd volume of 3'-ligation reaction with 7 units T4 RNA Ligase 1 (NEB, M0437M), 1 pmol preA-L3-irCLIP adaptor, and 20% PEG400 in 1x 3'-ligation buffer (50 mM Tris pH 7.5, 10 mM $MgCl_2$, 5 mM DTT) to the T4 PNK reaction. The 3'-ligation reaction was then briefly vortexed to mix well and resuspend the beads before incubated overnight incubation in a thermomixer at 16°C shaking 15s 1,400 RPM, 90s rest. The T4 PNK and 3'-ligation reactions were prepared as master mixes and distributed across all reactions. The following morning, the 3'-ligation reactions were removed and the immunoprecipitates were eluted using 10 µL of 1x LDS sample buffer (ThermoFisher, NP0007) in NT2 heating for 10 min at 75°C. Samples were centrifuged at 10,000g for 30s to pool eluates and then the total volume

of sample was immediately run out on a 4-12% Bis-Tris SDS-PAGE Gel (BioRad, 3450125) at 180V for 65 min in 1x MOPS Buffer. PAGE-resolved RNP complexes were imaged in-gel with an Odyssey CLx Imager (LI-COR) visualizing ligated RNA in the 800 channel. Target proteins should run ~15 kDa above expected MW (RNA fragment+preA-L3-800 adaptor).

Immunoprecipitation for Mass-Spectrometry

Up to 5 µg of antibody was loaded onto magnetic Protein G Dynabeads (ThermoFisher, 10004D) at 30 µg Ab /100 µL Protein G beads by incubation in 1x PBS + 0.02% Tween-20 at ambient temperature for 30 min with rotation. Mouse host primary antibodies were complexed with ½ equivalent of rabbit anti-mouse IgG Fc secondary (ThermoFisher, PI31194) whereas other host IgGs were complexed directly onto Protein G beads. Proteins were immunoprecipitated overnight at 4°C with end-over-end rotation. Immunoprecipitations were centrifuged briefly at 2,000g at 4°C to collect lysate. Lysates were removed from immunoprecipitates and set aside in labeled microcentrifuge tubes. Immunoprecipitates were then washed sequentially for 10 min each at 4°C with an equal volume of High-Stringency Buffer (20 mM Tris pH 7.5, 120 mM NaCl, 5 mM EDTA, 1% Trition-X100, 1% Na-deoxycholate, 0.001% SDS), High-Salt Buffer (20 mM Tris, pH 7.5; 1 M NaCl, 5 mM EDTA, 1% Trition-X100, 1% Sodium deoxycholate, 0.001% SDS), Low-Salt Buffer (20 mM Tris pH 7.5, 5 mM EDTA) and half-volume of NT2 buffer (50 mM Tris pH 7.5, 150 mM NaCl, 1 mM $MgCl_2$, 0.0005% IGEPAL CA-630). Immunoisolates were isolated via elution with 5% SDS in 100 mM Tris pH 7.5 at 70°C for 10 min. Eluates were subjected to an S-Trap Micro (Protifi, C02-micro-80) workflow digesting with 1 µg of sequencing grade Trypsin (Promega, V5113) per 10 µg of eluate (by BCA).

Mass-Spectrometry

In-Gel Digest. Triplicate samples from irCLIP were electrophoresed in a 4-12% Bis-Tris SDS-PAGE Gel (BioRad, 3450125) at 180V for 65 min in 1x MOPS Buffer. Following imaging, regions of each sample containing RNA signal (IR800) were excised with a razor blade and placed in a 1.5 mL Eppendorf tube. For the IgG control, the MW-range corresponding to anti-LGALS3 RNA associated signal was excised (>40 kDa). Gel slices were rinsed once each with 250 µL of Mili-Q water, acetonitrile, and 50 mM

ammonium bicarbonate at room-temperature for 20 min. Then, ensuring the gel slices were completely submerged, the samples were reduced for 30 min at 66°C in 5 mM DTT 50 mM Ammonium Bicarbonate. The reduced samples were alkylated with 25 mM iodocateamide (Sigma A3221-10VL). The slices were then rinsed with 50 mM ammonium bicarbonate and then 1:1 mixture of 50 mM ammonium bicarbonate:acetonitrile and dried by SpeedVac. The dried slices were resuspended in 0.1 µg Trypsin (Promega V5113)/ 200 µL 50 mM ammonium bicarbonate and permitted to digest overnight at 37°C. Then, the trypsin supernatant was removed and transferred to a 1.5 mL Eppendorf tube. To completely extract the peptides, the gel was extracted with thrice with 100 µL 70% acetonitrile + 1% formic acid in HPLC grade water (Fisher W5-4). The extractions were pooled with trypsin supernatant and concentrated overnight by SpeedVac. Then, the peptide sample was resuspended in 2.5% formic acid. Peptides were desalted with HyperSep C18 96-Well Filter Plate 5-7 µL bed volume (ThermoFisher, 60110-401).

Peptides were separated over a 25 cm EasySpray reverse-phase LC column (75 µm inner diameter packed with 2 µm, 100 Å, PepMapC18 particles, Thermo Fisher Scientific). The mobile phases (A: water with 0.2% formic acid and B: acetonitrile with 0.2% formic acid) were driven and controlled by a Dionex Ultimate 3000 RPLC nano system (Thermo Fisher Scientific). An integrated loading pump was used to load peptides onto a trap column (Acclaim PepMap 100 C18, 5 um particles, 20 mm length, Thermo Fisher Scientific) at 5 µL/minute, which was put in line with the analytical column 5.5 minutes into the gradient. The gradient flowed isocratically from at 0% B for the first 6 minutes of the analysis, followed by an increase from 0% to 5% B from 6 and 6.5 minutes, an increase from 5 to 25% B from 6.5 to 66 minutes, an increase from 25% to 90% from 66 to 70 minutes, isocratic flow at 90% B from 70-75 minutes, and a re-equilibration at 0% for 15 minutes for a total analysis time of 90 minutes. The eluate was ionized using an EASY-Spray ionization source (Thermo Fisher Scientific) held at +2.2 kV , the column was held at 40 °C, and the inlet capillary temperature was held at 275°C on the Orbitrap Fusion (Thermo Fisher Scientific) that was used for all LC-MS/MS analyses. Survey scans of peptide precursors were collected in the Orbitrap from m/z 300-1350 with an AGC target setting of 250% (1,000,000 charges), a

maximum injection time of 50 ms, RF lens at 60%, and a resolution of 60,000 at m/z 200. Monoisotopic precursor selection was enabled for peptide isotopic distributions, and precursors of z = 2-5 were selected for data-dependent MS/MS scans for 2 seconds of cycle time. Dynamic exclusion was set to exclude precursors after being selected once for an exclusion time of 30 seconds with a ±10 ppm window set around the precursor monoisotope. An isolation window of 1 m/z was used to select precursor ions with the quadrupole, and precursors were fragmented using a normalized HCD collision energy of 30. MS/MS scans were collected with an AGC target of 200% (100,000 charges), with a maximum accumulation time of 54 ms, an Orbitrap resolution of 30,000 at m/z 200, and a first mass set at m/z 120. The same method was used for CLIP-MS, GeLC-MS/MS, and IP-MS experiments.
Raw data were processed using MaxQuant version 1.6.10.43 (Tyanova, Temu, and Cox 2016), and tandem mass spectra were searched with the Andromeda search algorithm (Cox et al. 2011). Oxidation of methionine and protein N-terminal acetylation were specified as variable modifications, while carbamidomethylation of cysteine was set as a fixed modification. Precursor ion search tolerances of 20 ppm and 4.5ppm were used for first and main searches, respectively, a product ion mass tolerance of 20 ppm were used for MS/MS scans, and two missed cleavages were allowed for full trypsin specificity.
Data Analysis. Peptide spectral matches were made against a target-decoy human reference proteome database downloaded from Uniprot (Elias and Gygi 2007). Peptides were filtered to a 1% FDR and a 1% protein FDR was applied according to the target-decoy method. Proteins were quantified and normalized using MaxLFQ (Cox et al. 2014) with a label-free quantification (LFQ) minimum ratio count of 1. LFQ intensities were calculated using the match between runs feature, and MS/MS spectra were required for LFQ comparisons. For quantitative comparisons, protein intensity values were log2-transformed before further analysis, and missing values were imputed from a normal distribution with width 0.3 and downshift value of 2.5 using the Perseus software suite (Tyanova, Temu, Sinitcyn, et al. 2016). Peptides were identified with MaxQuant label-free quantitation. Relative enrichment of log2-transformed intensities was assessed on a per-protein basis with an FDR computed by a Benjamini-Hochberg

adjusted t-test. Peptides were analyzed on a per-protein basis and plotted and annotated using the ggplot2 package in R (Wickham 2009).

Orthogonal Organic Phase Separation (OOPS)

Based on the method of Querioz and Villanueva (Queiroz et al. 2019; Villanueva et al. 2020)**.** Clarified whole-cell lysates from *UV Crosslinking* were thawed, assessed by BCA, and diluted to 2 mg/mL with 1x PBS. Then, 800 µL of Trizol (Trizol, Thermo Fisher Scientific) was added to 200 µg/100 µL of lysates in a 1.5 mL eppendorf tube. The mixture was incubated at room temperature (RT) for 5 min to dissociate unstable RNA–protein interactions. For biphasic extraction, 200 µL of chloroform (Fisher Scientific) were added, phases were vortexed, and the sample was centrifuged for 15 min at 18,000g at 4°C. The upper, aqueous phase (containing non-cross-linked RNAs) was transferred to a new tube, froze, and stored for future use. The lower, organic phase (containing non cross-linked proteins) was transferred to a new tube and proteins were precipitated by addition of nine volumes of LC-MS grade methanol (Fisher Scientific A456-4). The remaining interface (approx. 100 µL), containing the protein–RNA crosslinks, was twice extracted with 900 µL Trizol and 200 µL chloroform. For each extraction, the aqueous and organic phases (upper and lower) were discarded, preserving the interphase. Then, the interphase was precipitated by addition of nine volumes of -20°C LC-MS grade methanol, incubated on dry ice for minimum 60 min, and pelleted by centrifugation at 18,000g at 4°C for 10 min.

For RNA-binding protein analyses, the precipitated interface was resuspended in 100 µL of 100mM Tris pH 7.5, 1 mM $MgCl_2$, 1% SDS, incubated at 95 °C for 5 min, cooled. The sample was split and half was digested with 1 µg RNase A (ThermoFisher, EN0531) for 2h at 37°C. Then, 10 µL of each sample was diluted with 4x LDS sample buffer (ThermoFisher, NP0007) and run out for Immunoblot. Bands appearing in RNase A treated samples, but absent in untreated samples correspond to RNA-protein crosslinks of the probed protein.

Immunoblot

Samples were run out on a 4-12% Bis-Tris SDS-PAGE Gel (BioRad, 3450125) at 180V for 65 min in 1x MOPS Buffer (BioRad, 1610788). Proteins were transferred onto 0.2 µm nitrocellulose membranes (BioRad, 1704271) using the Transblot Turbo System

(BioRad, 1704150). Membranes were blocked in 1x Blocking Buffer (1x PBS pH 7.4, 0.05% Tween-20, 5% Bovine Serum Albumin) for 1h at room temperature with gentle agitation. Then, the membrane was washed thrice with PBS-T (1x PBS pH 7.4 + 0.05% Tween-20) allowing the membrane to shake for 5 min. Primary antibodies targeting the proteins were diluted in 1x Blocking Buffer and incubated overnight at 4°C with gentle agitation. Following incubation in primary, the membranes were washed thrice with 1x PBS-T 0.05% for 5 min at room temperature. Then, a dilution of the appropriate secondary was used to detect antibody binding. Finally, the blots were imaged with an Odyssey CLx Imager (LI-COR).

Immunofluorescence Microscopy

Adherent HeLa cells were seeded at 1E4 cells/well in an 8-well chamber slide and grown for 48 hours. Then, the cells were then washed thrice with ice-cold 1x DPBS pH 7.4, fixed with 4% Paraformaldehyde (ThermoFisher, 28906) for 15 min at room temperature. The fixed cells were then washed 2x with ice-cold 1x DPBS before permeabilization with 0.1% Triton X-100 in 1x PBS pH 7.4 for 10 min at room temperature. Then, the fixed and permeabilized cells were washed 2x with ice-cold 1x PBS before blocking in a solution of 10% Goat Serum (Gibco/ThermoFisher, 16210064) in 1x PBS overnight at 4°C with gentle agitation. Following removal of the blocking solution, the wells were washed 3x with ice-cold 1x PBS and incubated with primary antibodies at the desired concentrations (2.5 μg/mL) in 1x PBS + 10% Goat Serum overnight at 4°C with gentle agitation. Finally, the cells were washed 3x with ice-cold PBS before addition of secondary antibodies at 1 μg/mL and DAPI at μg/mL 1 in 1x PBS + 10% Goat Serum for 1 hour at room-temperature with gentle agitation. After removal of the secondary, the cells were washed 3x with 1x PBS. Cells were then mounted with Vectashield anti-fade mounting medium (Vector Laboratories, H-1000-10) and allowed to cure overnight at room temperature after sealing coverslip. All incubation steps after introduction of fluorescent imaging reagents were conducted in the dark. After mounting, slides were imaged at 60x oil-immersion on an Nikon A1R confocal microscope.

Lactose Affinity Chromatography

Whole-cell lysates from HeLa were prepared using a modified 1x RIPA (20 mM Tris HCl, 200 mM NaCl, 1.0% (v/v) IGEPAL CA-630, 0.5% (w/v) Sodium Deoxycholate, 1.0 mM EDTA, a pH of 7.5) with 1x Mammalian ProteaseArrest™ (G Biosciences, #786-331). Lysates were normalized to 2 mg/mL. Using an ÄKTA FPLC (GE), 1000 µg of lysate was loaded onto a 1 mL column of lactosyl-agarose (Sigma-Aldrich, L7634-5ML) pre-equilibrated with lactose-affinity buffer (20 mM Tris pH 7.5, 200 mM NaCl, 2.5 mM $MgCl_2$, 0.1 mM lactose, 1 mM TCEP). After loading, the column was washed with 5 column volumes of lactose-affinity buffer. Then, galectin-3 was eluted on a 10 column volume gradient from 0.1 mM lactose to 25 mM using a matched elution buffer (20 mM Tris pH 7.5, 200 mM NaCl, 2.5 mM $MgCl_2$, 25 mM lactose, 1 mM TCEP). Fractions were were analyzed by SDS-PAGE immunoblot to detect presence of galectins.

Supplementary Methods Table I. Antibody clones and product numbers. sgRNA sequences. PCR and Sequencing Oliogs. HDR Template sequence.

Abbreviations:

RBP – RNA Binding Protein

irCLIP – infrared visualized UV-Crosslinking and Immunoprecipitation

HDR – Homology Directed Repair

3'UTR – 3' Untranslated Region

HA-tag – influenza hemagglutinin derived tag (YPYDVPDYA)

OOPS – orthogonal organic phase separation

Figure S1: Anti-Galectin-3 irCLIP in 4T1 and MDA-MB-231

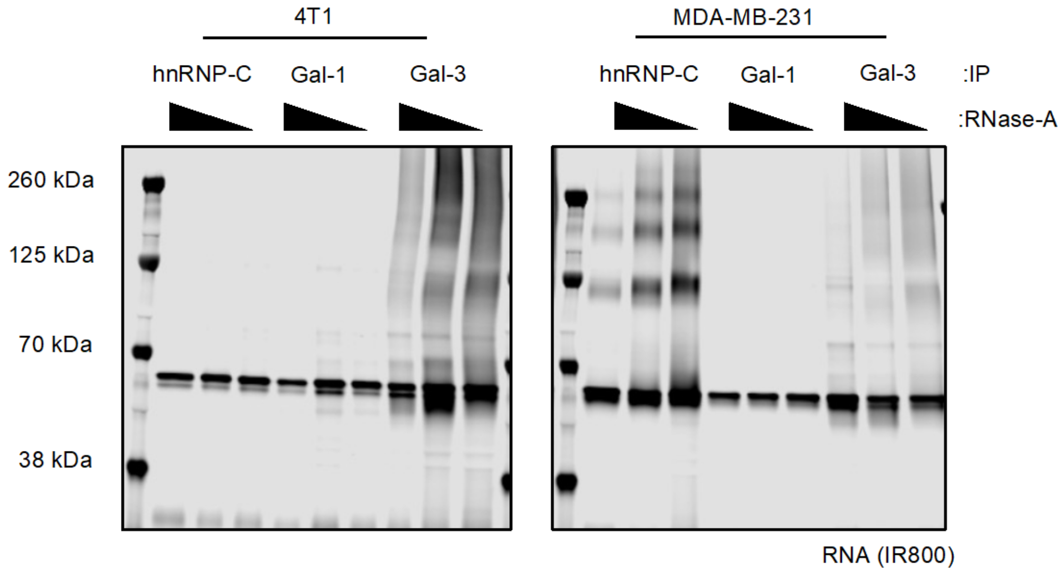

Figure S1 Legend:

Anti-galectin-3 irCLIP identifies RNA signal in the breast cancer lines 4T1 (murine) and MDA-MB-231 (human).

Figure S2: Anti-Galectin-3 irCLIP in MRC5

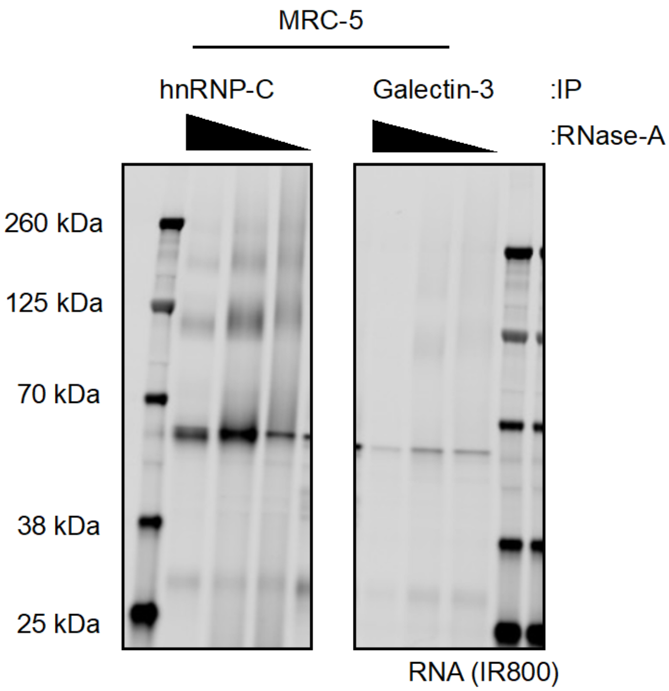

Figure S2 Legend:

Anti-Galectin-3 irCLIP does not detect RNA signal in MRC5 cells.

Figure S3: anti-Galectin-3 IP-MS in Nucleus and Cytosol

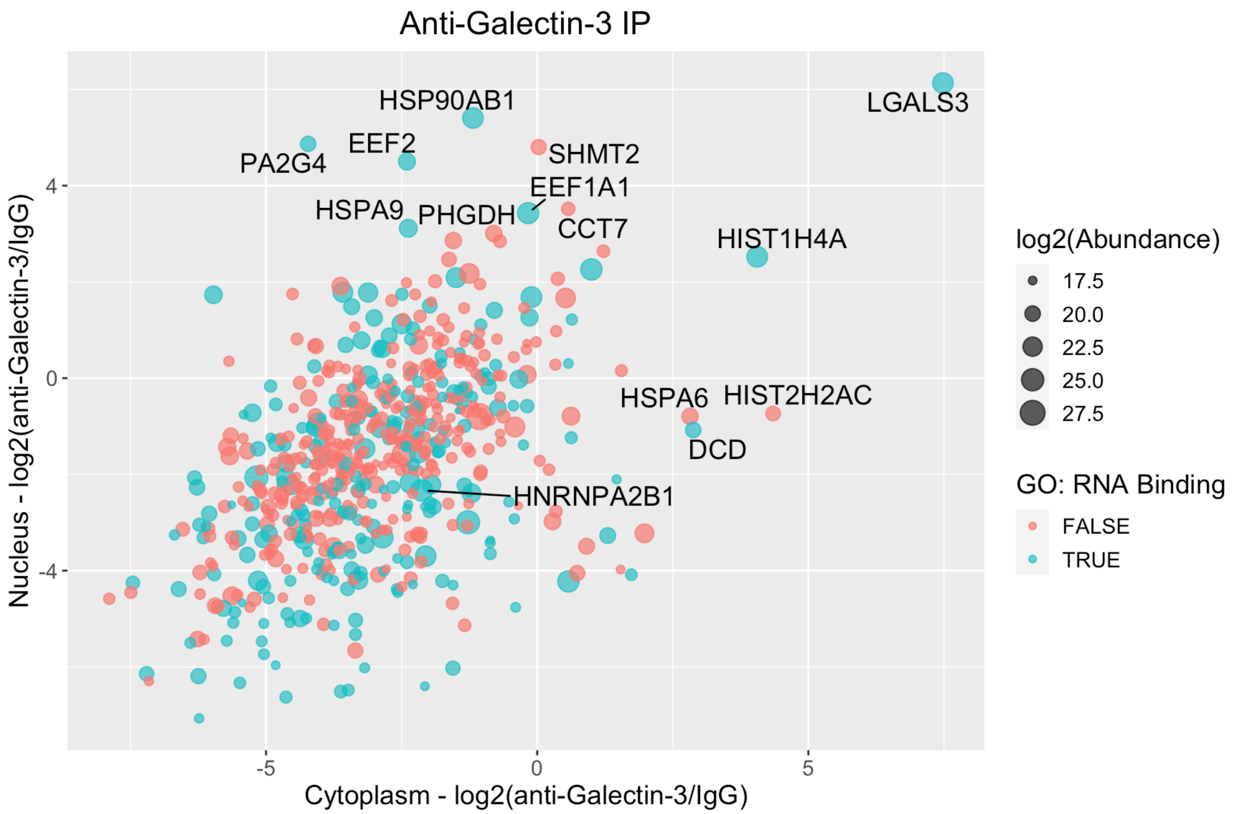

Figure S3 Legend:

Anti-Galectin-3 [EPR19244] IP-MS in Nuclear and Cytoplasmic fractions of HeLa. In both the Nuclear and Cytoplasmic fractions, galectin-3 (LGALS3) is the most highly enriched protein.

Figure S4: Relative abundance of identified proteins in irCLIP-MS

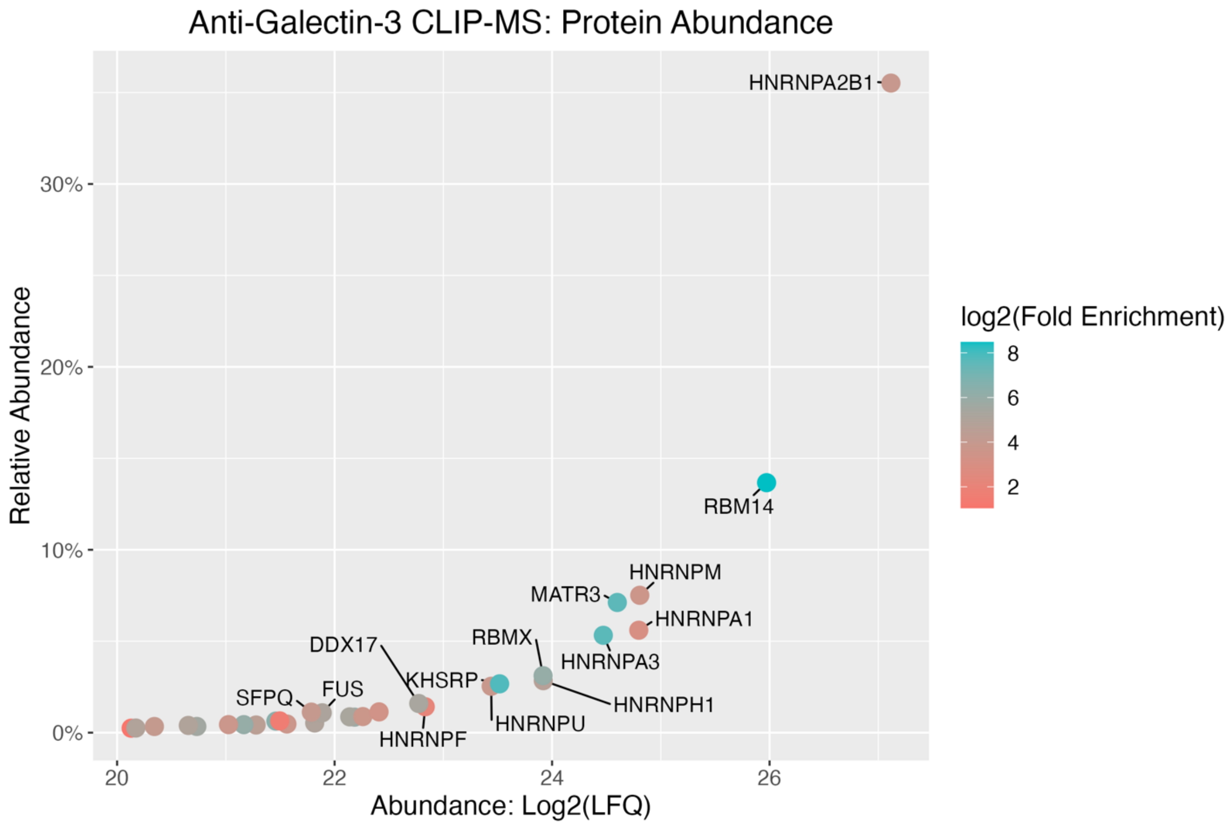

Figure S4 Legend:

Observed absolute and relative abundance of proteins in anti-Galectin-3 CLIP-MS samples (in-gel digest). hnRNPA2B1 is the most abundant protein, but is not the most highly enriched.

Figure S5: Galectin-3 and hnRNPA2B1 - Low-complexity domain homology

>LEG3_HUMAN Galectin-3 (N-Terminal Domain)
MADNFSLHDALSGSGNPNPQGWPGAWGNQPAGAGGYPGASYPGAYPGQAPPGAYPGQAPP
GAYPGAPGAYPGAPAPGVYPGPPSGPGAYPSSGQPSATGAYPATGPYGAPAGPLIVPYNL
PLPGGVVPR

>ROA2_HUMAN Heterogeneous nuclear ribonucleoproteins A2/B1 (Low Complexity Domain)
RKALSRQEMQEVQSSRSGRGGNFGFGDSRGGGGNFGPGPGSNFRGGSDGYGSGRGFGDGY
NGYGGGPGGGNFGGSPGYGGGRGGYGGGGPGYGNQGGGYGGGYDNYGGGNYGSGNYNDFG
NYNQQPSNYGPMKSGNFGGSRNMGGPYGGGNYGPGGSGGSGGYGGRSRY

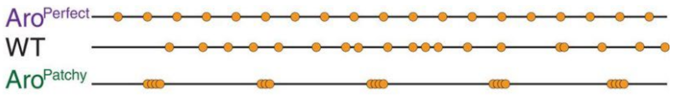

Figure S5 Legend:

Comparison of Galectin-3 (N-terminal domain) and hnRNPA2B1 (Low-complexity domain). Aromatic residues are highlighted (Phenylalanine/Tryptophan – purple, Tyrosine – red). The two protein fragments share similarity with respect to periodic spacing of aromatic residues in-between flexible and proline. This may contribute to cross-recognition between some anti-galectin-3 antibodies and hnRNPA2B1.

Figure S6: Galectin-3 and hnRNPA2B1 - HeLa CRISPR-KO clone validation

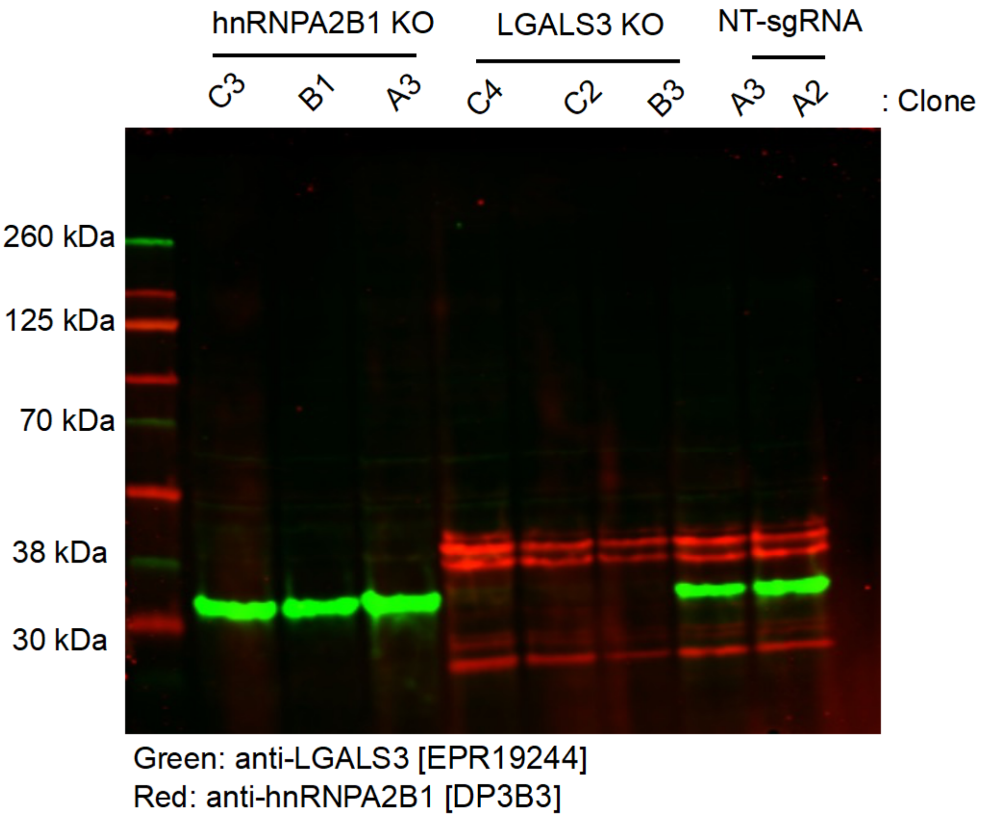

Figure S6 Legend:

Immunoblot verification of clonal galectin-3 and hnRNPA2B1 knockout.

Figure S7: OOPS Workflow Schematic - from Villanueva et al. 2020

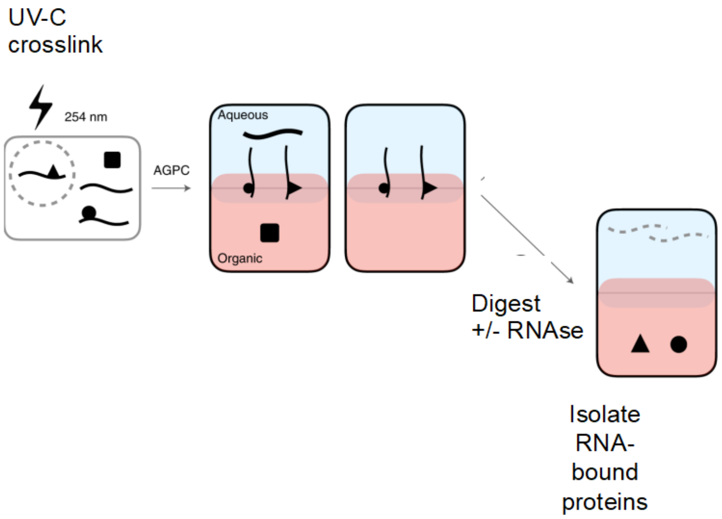

Figure S7 Legend:

Overview of orthogonal organic phase separation from Villuneva et al. (2020). UV-crosslinked RBPs are extracted by sequential extraction of the interface layer with Trizol. RBPs are assessed via immunoblot +/- RNAse treatment with appearance of protein band following RNA treatment indicating an RNA-associated protein.

Figure S8: OOPS with anti-Galectin-3 and anti-hnRNPA2B1 (Overlay)

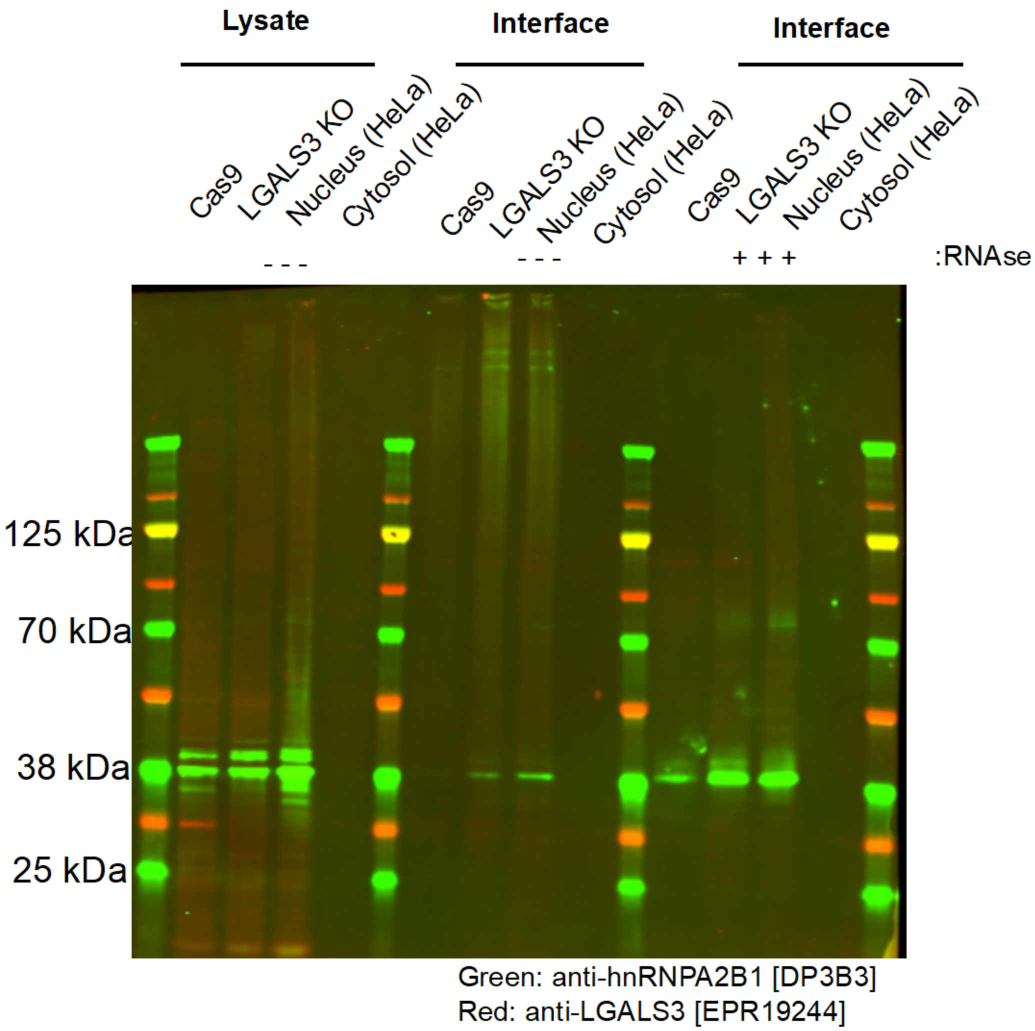

Figure S8 Legend:

OOPS of Cas9-NT-sgRNA, LGALS3 KO, Nuclear, or Cytosolic fractions. Two color immunoblot with anti-galectin-3 and anti-hnRNPA2B1.

Figure S9: OOPS with anti-Galectin-3 and anti-hnRNPA2B1 (Side-by-Side)

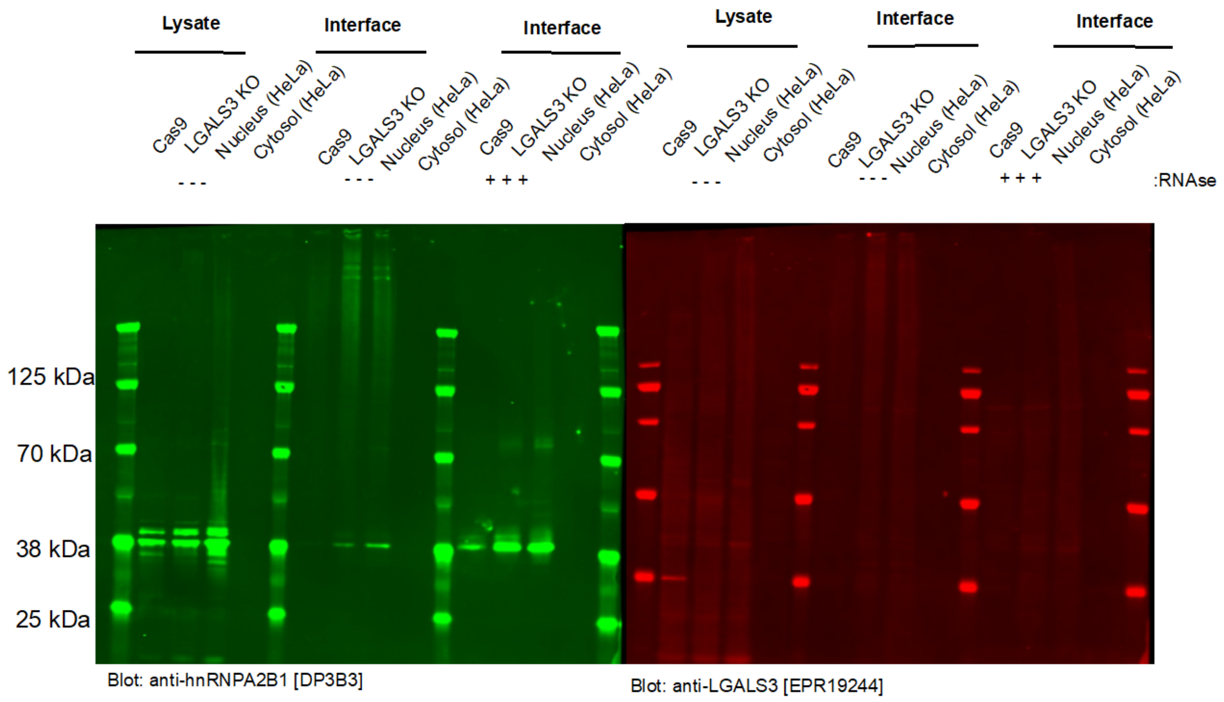

Figure S9 Legend:

Single color Immunoblots from Figure S8.

Figure S10: Lactose affinity purification of LGALS3 and LGALS3-HA

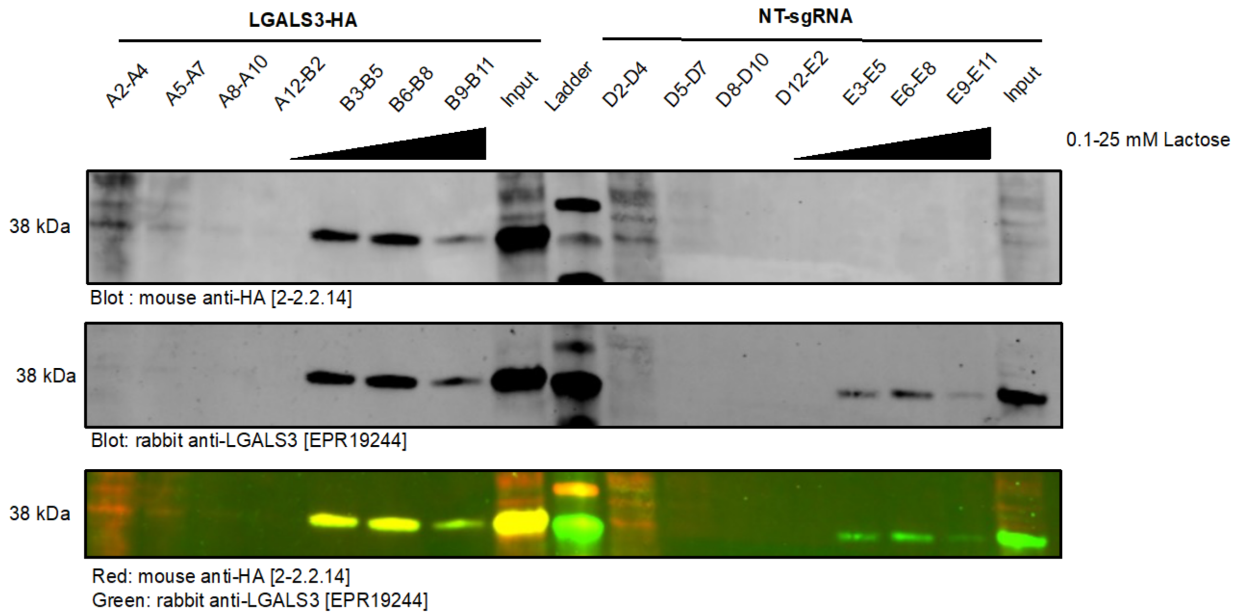

Figure S10 Legend:

Lactose affinity purification of galectin-3 and LGASL3-HA from HeLa via Fast-Protein Liquid Chromatography. Whole-cell lysate from HeLa is passed over a 1 mL lactosyl-agarose column (LGALS3-HA: A2-A4; NT-sgRNA: D2-D4), washed (LGALS3-HA: A5-A10; NT-sgRNA: D5-D10), and eluted with a lactose gradient (0.1-25 mM lactose; LGALS3-HA: A12-B11; NT-sgRNA: D12-E11). Fractions were analyzed by immunoblot using an anti-LGALS3 [EPR19244] and anti-HA [2-2.2.14] to verify the elution of galectin-3 and LGALS3-HA following the introduction of lactose to the buffer system.

Figure S11: Immunofluorescence microscopy of LGALS3 in HeLa at 60x objective

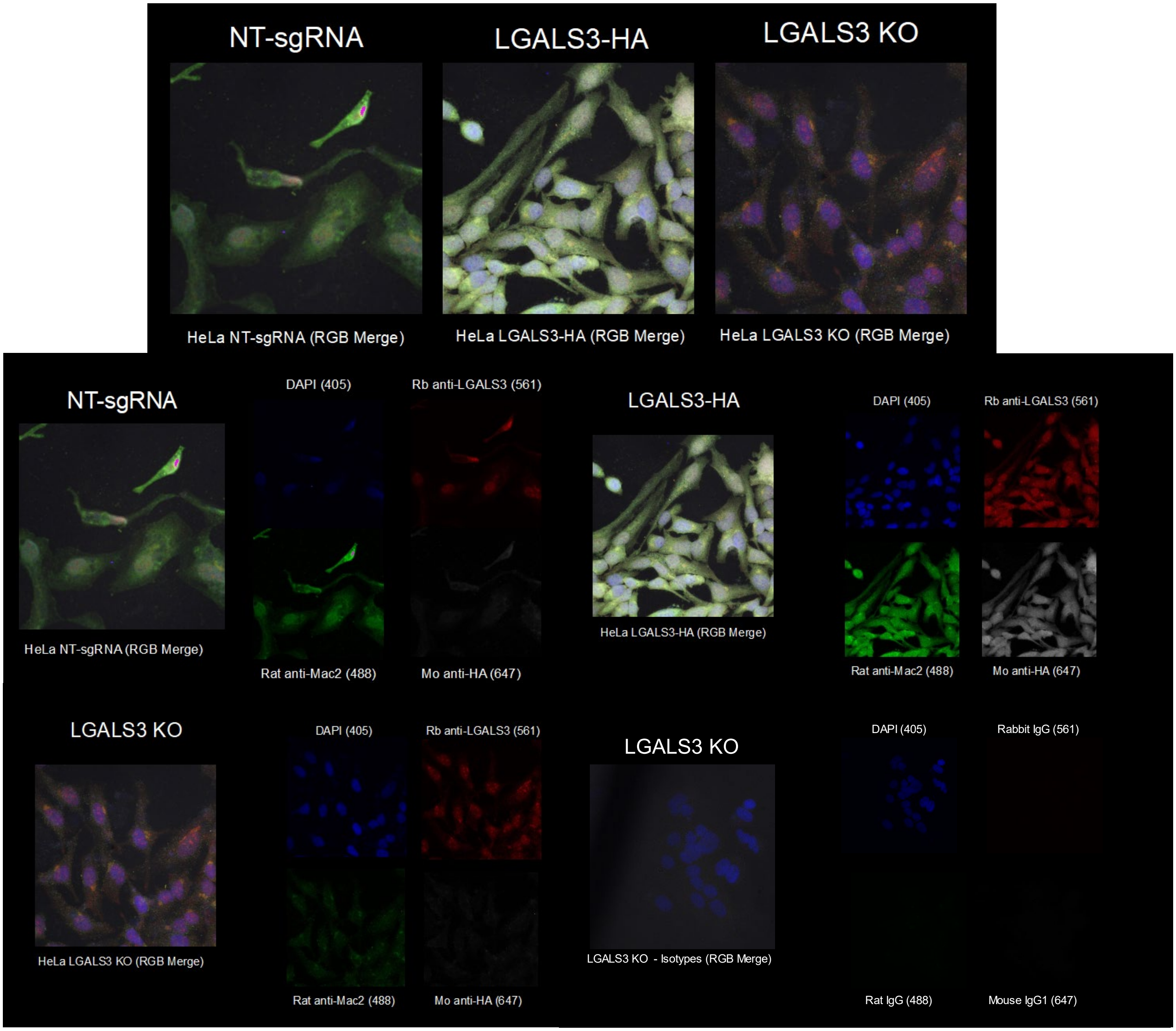

Figure S11 Legend:
Immunofluorescence staining of LGALS3 in HeLa via confocal microscopy at a 60x objective. Wild-type NT-sgRNA background shows diffuse nucleocytosolic staining. Staining with rat anti-galectin-3 [Mac2] mAb and rabbit anti-LGALS3 [EPR19422] was attenuated by CRISPR-Cas9 knock-out of LGALS3, retaining some residual staining that co-localizes with the nuclear marker DAPI. In the LGALS3-HA background, diffuse nucleocytosolic staining was observed with all antibody probes, including mouse anti-HA [2-2.2.14]. Minimal background staining was observed with isotype-matched controls.

Figure S12: IP-MS Characterization of anti-LGALS3 [EPR19244]

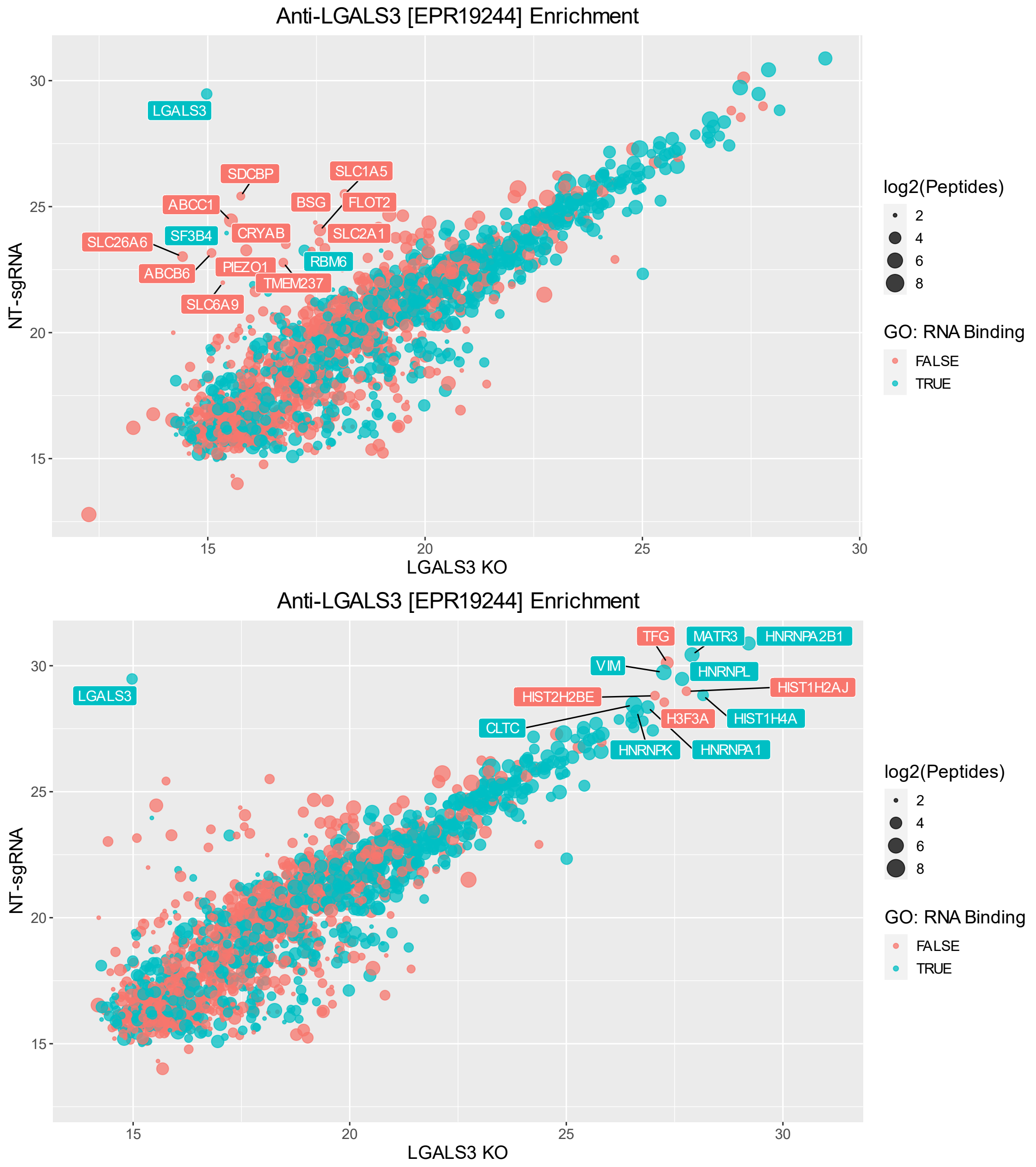

Figure S12 Legend:

IP-MS of anti-LGALS3 [EPR19244]. Comparison of Log2(LFQ Intensities) in NT-sgRNA and LGALS3 KO backgrounds from IP of whole-cell lysates. Proteins with Gene Ontology RNA-binding function annotated are highlighted in blue. (Top) Proteins enriched in NT-sgRNA relative to LGALS3 KO. (Bottom) Proteins present at high abundance in both NT-sgRNA and LGALS3 KO.

Figure S13: IP-MS Characterization of anti-LGALS3 [Mac2]

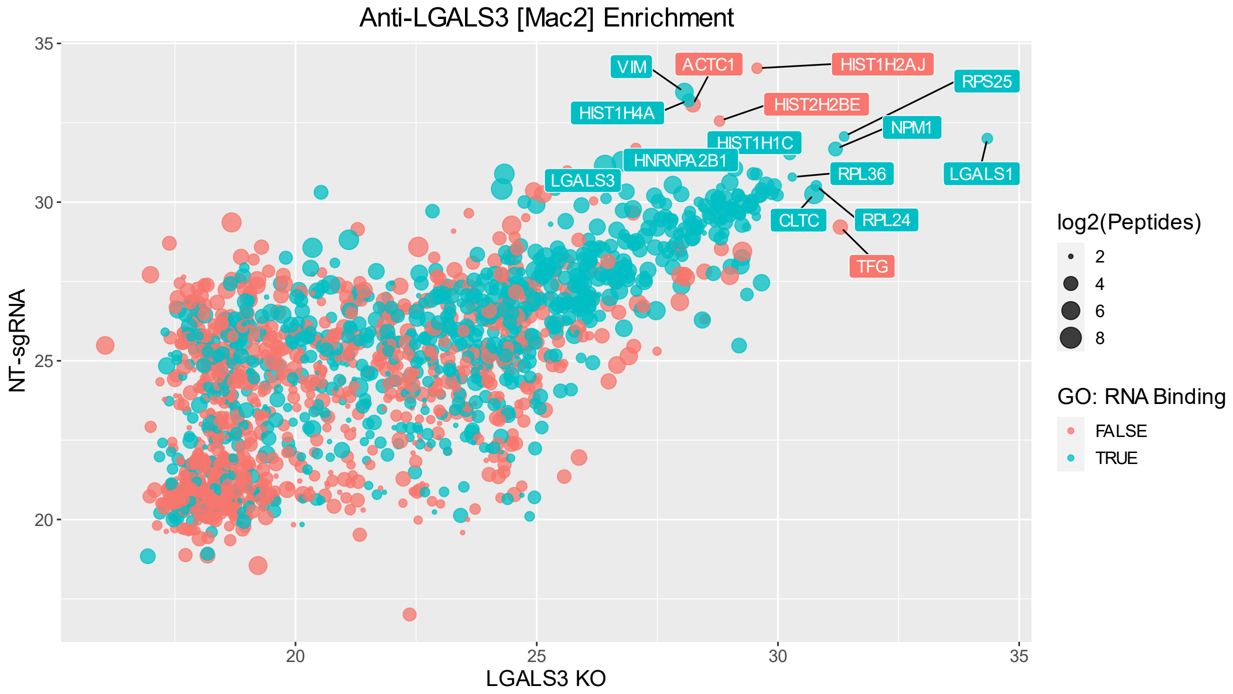

Figure S13 Legend:

IP-MS of anti-LGALS3 [Mac2]. Comparison of Log2(LFQ Intensities) in NT-sgRNA and LGALS3 KO backgrounds from IP of whole-cell lysates. Proteins with Gene Ontology RNA-binding function annotated are highlighted in blue. Labeled: LGALS3 and Proteins strongly enriched in both NT-sgRNA and LGALS3 KO conditions. Note: the Mac2 clone appears to enrich LGALS1 in the absence of LGALS3.

Figure S14: IP-MS Characterization of anti-LGALS3 [A3A12]

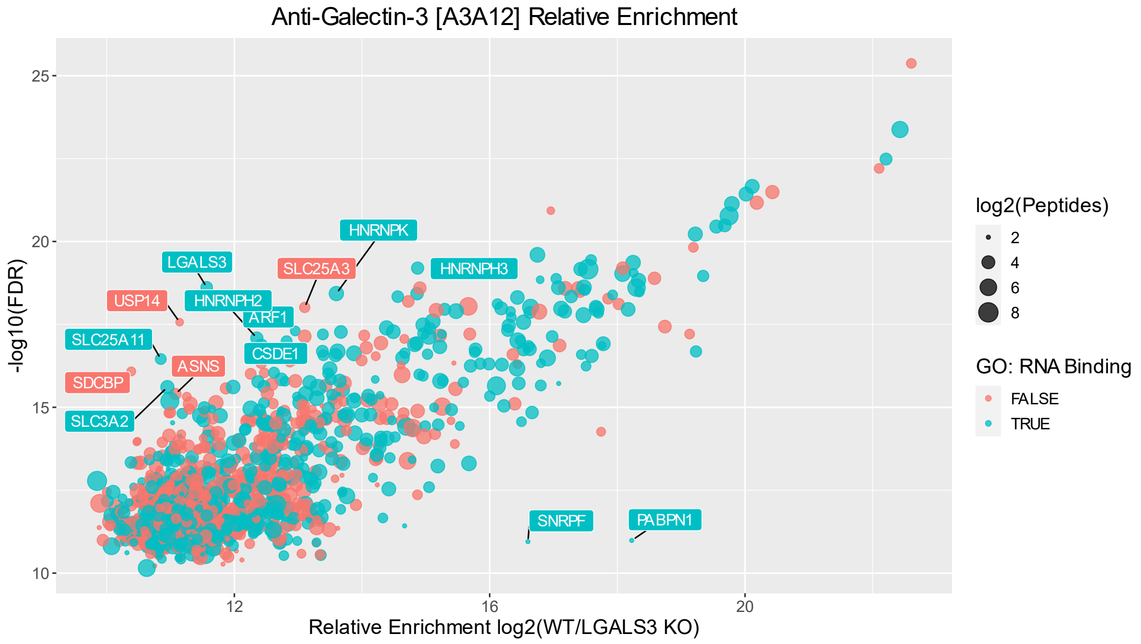

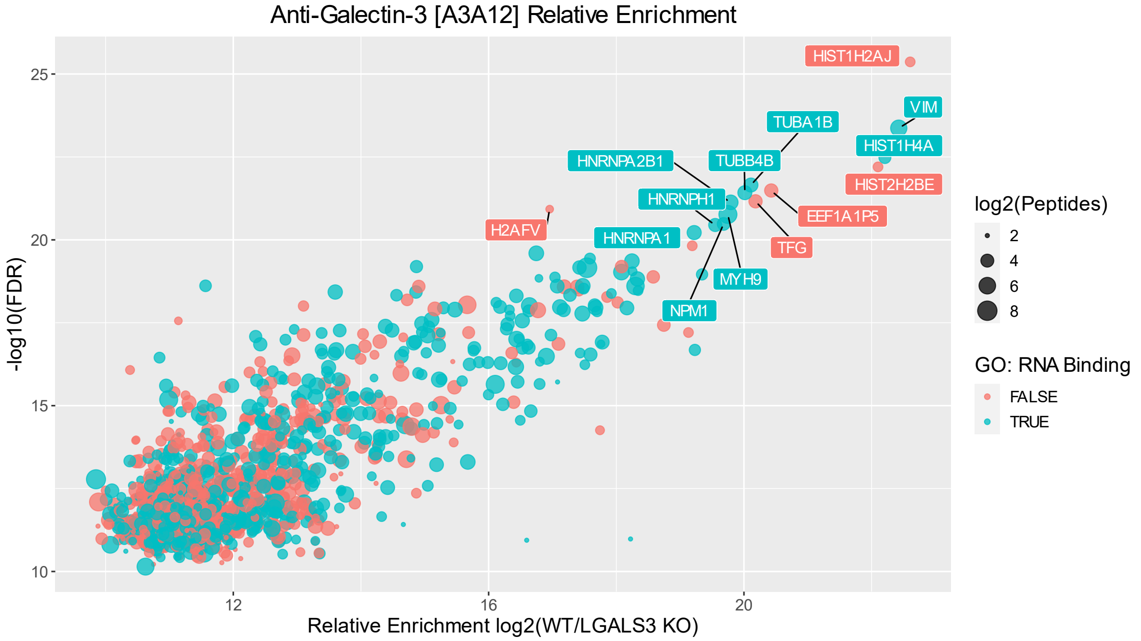

Figure S14 Legend:

IP-MS of anti-LGALS3 [A3A12]. Comparison of Log2(LFQ Intensities) in NT-sgRNA and LGALS3 KO backgrounds from IP of whole-cell lysates. Proteins with Gene Ontology RNA-binding function annotated are highlighted in blue. (Top) Proteins enriched in NT-sgRNA relative to LGALS3 KO. (Bottom) Proteins present at high abundance in both NT-sgRNA and LGALS3 KO.

Figure S15: LGALS3 dependent enrichment of anti-LGALS3 mAbs

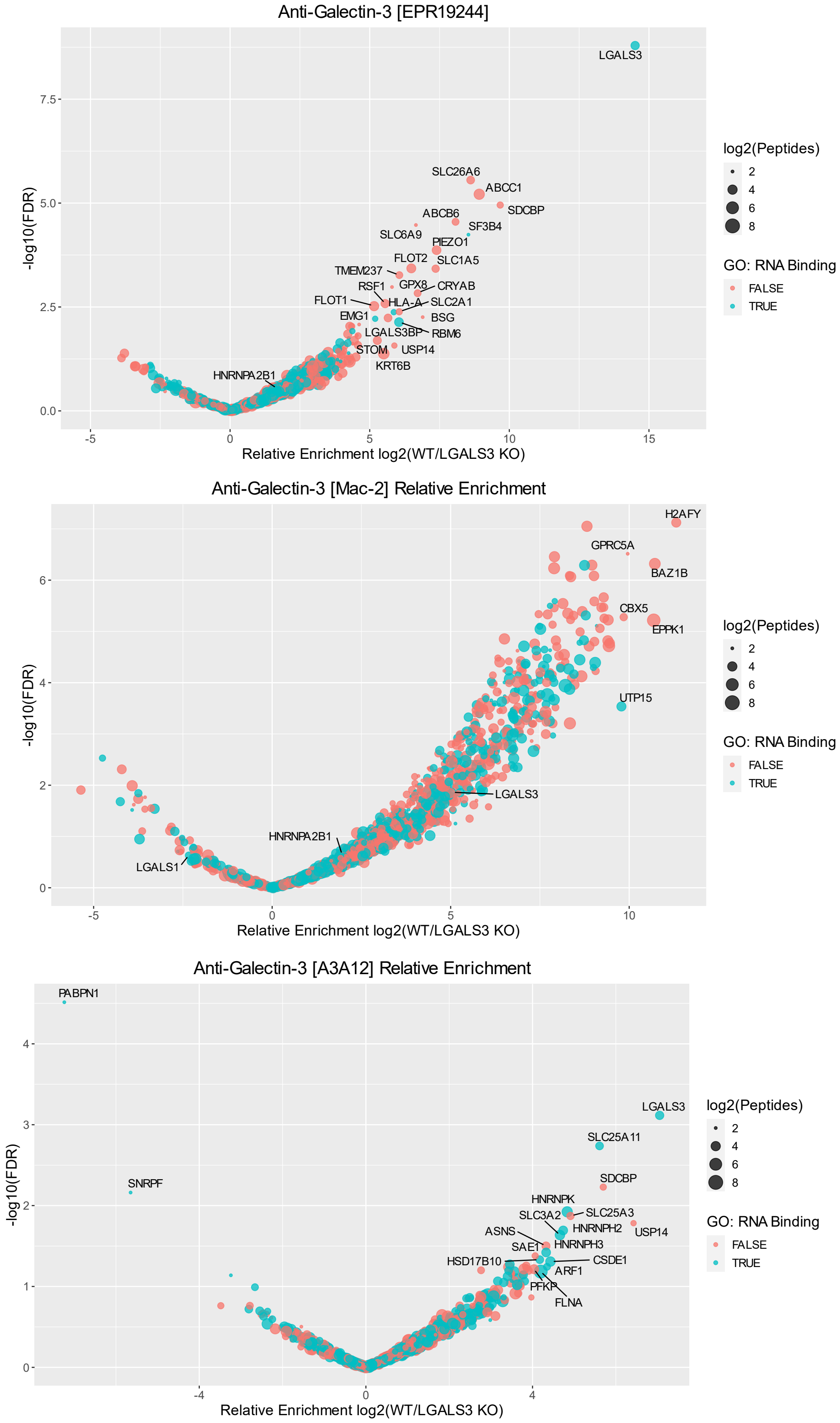

Figure S15 Legend:
Relative enrichment of anti-Galectin-3 mAbs in NT-sgRNA (WT) and LGALS3 KO HeLa. Comparison of Log2(LFQ Intensitiy) from immunoprecipitation of whole-cell lysates. Proteins with Gene Ontology RNA-binding function annotated are highlighted in blue. (Top) Differential enrichment of anti-LGALS3 [EPR19244] in NT-sgRNA and LGALS3 KO. (Middle) Differential enrichment of anti-LGALS3 [Mac2] in NT-sgRNA and LGALS3 KO. (Bottom) Differential enrichment of anti-LGALS3 [A3A12] in NT-sgRNA and LGALS3 KO. All mAbs enrich LGALS3, but to different degrees and with distinct backgrounds, co-enriching Gene Ontology annotated RNA-binding proteins, including some well characterized RNA-binding proteins such as SNRNPF [A3A12], and non-RNA-binding proteins.

Supplementary References:

Cho NH, Cheveralls KC, Brunner A-D, Kim K, Michaelis AC, Raghavan P, Kobayashi H, Savy L, Li JY, Canaj H, et al. 2022. OpenCell: Endogenous tagging for the cartography of human cellular organization. Science. 375(6585):eabi6983. doi:10.1126/science.abi6983.

Cox J, Hein MY, Luber CA, Paron I, Nagaraj N, Mann M. 2014. Accurate Proteome-wide Label-free Quantification by Delayed Normalization and Maximal Peptide Ratio Extraction, Termed MaxLFQ *. Mol Cell Proteomics. 13(9):2513–2526. doi:10.1074/mcp.M113.031591.

Cox J, Neuhauser N, Michalski A, Scheltema RA, Olsen JV, Mann M. 2011. Andromeda: A Peptide Search Engine Integrated into the MaxQuant Environment. J Proteome Res. 10(4):1794–1805. doi:10.1021/pr101065j.

Elias JE, Gygi SP. 2007. Target-decoy search strategy for increased confidence in large-scale protein identifications by mass spectrometry. Nat Methods. 4(3):207–214. doi:10.1038/nmeth1019.

Gagnon KT, Li L, Janowski BA, Corey DR. 2014. Analysis of nuclear RNA interference in human cells by subcellular fractionation and Argonaute loading. Nat Protoc. 9(9):2045–2060. doi:10.1038/nprot.2014.135.

Lin S, Staahl BT, Alla RK, Doudna JA. 2014. Enhanced homology-directed human genome engineering by controlled timing of CRISPR/Cas9 delivery. Weigel D, editor. eLife. 3:e04766. doi:10.7554/eLife.04766.

Queiroz RML, Smith T, Villanueva E, Marti-Solano M, Monti M, Pizzinga M, Mirea D-M, Ramakrishna M, Harvey RF, Dezi V, et al. 2019. Comprehensive identification of RNA–protein interactions in any organism using orthogonal organic phase separation (OOPS). Nat Biotechnol. 37(2):169–178. doi:10.1038/s41587-018-0001-2.

Tyanova S, Temu T, Cox J. 2016. The MaxQuant computational platform for mass spectrometry-based shotgun proteomics. Nat Protoc. 11(12):2301–2319. doi:10.1038/nprot.2016.136.

Tyanova S, Temu T, Sinitcyn P, Carlson A, Hein MY, Geiger T, Mann M, Cox J. 2016. The Perseus computational platform for comprehensive analysis of (prote)omics data. Nat Methods. 13(9):731–740. doi:10.1038/nmeth.3901.

Uphoff CC, Drexler HG. 2005. Detection of Mycoplasma Contaminations. In: Helgason CD, Miller CL, editors. Basic Cell Culture Protocols. Totowa, NJ: Humana Press. (Methods in Molecular Biology™). p. 13–23. [accessed 2023 Apr 8]. https://doi.org/10.1385/1-59259-838-2:013.

Villanueva E, Smith T, Queiroz RML, Monti M, Pizzinga M, Elzek M, Dezi V, Harvey RF, Ramakrishna M, Willis AE, et al. 2020. Efficient recovery of the RNA-bound proteome and protein-bound transcriptome using phase separation (OOPS). Nat Protoc. 15(8):2568–2588. doi:10.1038/s41596-020-0344-2.

Wickham H. 2009. ggplot2: Elegant Graphics for Data Analysis. New York, NY: Springer New York. [accessed 2023 Apr 14]. https://link.springer.com/10.1007/978-0-387-98141-3.

Zarnegar BJ, Flynn RA, Shen Y, Do BT, Chang HY, Khavari PA. 2016. irCLIP platform for efficient characterization of protein–RNA interactions. Nat Methods. 13(6):489–492. doi:10.1038/nmeth.3840.
